# Genome-wide identification and characterization of caffeoyl-coenzyme A O-methyltransferase genes related to the Fusarium head blight response in wheat

**DOI:** 10.1186/s12864-021-07849-y

**Published:** 2021-07-04

**Authors:** Guang Yang, Wenqiu Pan, Ruoyu Zhang, Yan Pan, Qifan Guo, Weining Song, Weijun Zheng, Xiaojun Nie

**Affiliations:** 1 State Key Laboratory of Crop Stress Biology in Arid Areas, College of Agronomy and Yangling Branch of China Wheat Improvement Center, Northwest A&F University, 712100 Yangling, Shaanxi China; 2 ICARDA-NWSUAF Joint Research Centre, Northwest A&F University, 712100 Yangling, Shaanxi China

**Keywords:** CCoAOMT gene family, Fusarium head blight (FHB), Expression pattern, Regulation module, Genetic variation, Wheat

## Abstract

**Background:**

Lignin is one of the main components of the cell wall and is directly associated with plant development and defence mechanisms in plants, especially in response to *Fusarium graminearum* (Fg) infection. Caffeoyl-coenzyme A O-methyltransferase (CCoAOMT) is the main regulator determining the efficiency of lignin synthesis and composition. Although it has been characterized in many plants, to date, the importance of the CCoAOMT family in wheat is not well understood.

**Results:**

Here, a total of 21 wheat CCoAOMT genes (*TaCCoAOMT*) were identified through an *in silico* genome search method and they were classified into four groups based on phylogenetic analysis, with the members of the same group sharing similar gene structures and conserved motif compositions. Furthermore, the expression patterns and co-expression network in which *TaCCoAOMT* is involved were comprehensively investigated using 48 RNA-seq samples from *Fg* infected and mock samples of 4 wheat genotypes. Combined with qRT-PCR validation of 11 *Fg*-responsive *TaCCoAOMT* genes, potential candidates involved in the FHB response and their regulation modules were preliminarily suggested. Additionally, we investigated the genetic diversity and main haplotypes of these CCoAOMT genes in bread wheat and its relative populations based on resequencing data.

**Conclusions:**

This study identified and characterized the CCoAOMT family in wheat, which not only provided potential targets for further functional analysis, but also contributed to uncovering the mechanism of lignin biosynthesis and its role in FHB tolerance in wheat and beyond.

**Supplementary Information:**

The online version contains supplementary material available at 10.1186/s12864-021-07849-y.

## Background

Wheat is considered as one of the most important staple crops worldwide, which accounts for approximately 30 % of the global cultivated area, and provides 20 % of the world’s food for consumption [1, 2]. Wheat is also an important source of human protein and mineral element intake [3, 4]. Continuously increasing and stable production of wheat holds the promise for ensuring global food security under the challenge of population boom and climate change as well as limited resource input in the future [5]. Fusarium head blight (FHB), which is also called scab and is caused mainly by *Fusarium graminearum* (*Fg*), is one of the most destructive diseases of wheat, resulting in huge wheat yield loss and the imposition of substantial health threats in both humans and livestock due to the DON toxin [6, 7]. More concerning, FHB has gradually become the major hazard and limitation of wheat production in recent years because of climate change and the expansion of conservation agriculture [8]. Thus, revealing the mechanism underlying FHB resistance and breeding FHB-tolerant wheat varieties are crucial to address these problems.

The cell wall is mainly composed of polysaccharides, phenolic compounds and proteins. In plant cells, the cell wall always has mechanical and regulatory functions [9]. Extensive studies have reported that the components of the cell wall endow plants with the ability to resist the invasion of pathogens [10, 11]. Giancaspro et al. found that the pectin methylesterase PME-1 and β, 1–3 glucanase (Glu-1) could be considered putative causal genes of FHB resistance in wheat, as they are involved in cell wall metabolism and regulated the non-specific lipid transfer protein (nsLTP-1) [10]. Lionetti et al. showed that PMEIs (pectin methylesterase inhibitors) could dynamically regulate the PME (pectin methylesterase) activity during Botrytis infection, and *AtPMEI10*, *AtPMEI11* and *AtPMEI12* were verified to play the significant roles in cell wall metabolism to enhance plant immunity [12].

Lignin is the second most abundant component of the plant cell wall, and is also involved in the basal disease resistance in plants [13]. It was reported that lignin accumulated rapidly in the ear cell wall of both resistant and susceptible wheat spikes during *Fusarium culmorum* infection, and the content of lignin in resistant spikes was significantly higher than that in susceptible spikes [14]. Soni et al. found that silencing of *TaNAC032* could significantly decrease the lignin content in the rachis, resulting in the increased susceptibility to *Fg* infection in transgenic wheat [15]. Dhokane et al. integrated metabolo-transcriptomics to reveal the FHB candidate resistance gene in wheat QTL-Fhb2 and found that CAD (cinnamyl alcohol dehydrogenase) might be the putative resistance gene localized within the QTL-Fhb2 region, which is the crucial gene for lignin synthesis [16]. These studies suggested that lignin might also be involved in the FHB response and tolerance in wheat.

Lignin has been demonstrated to polymerize via dehydrogenation three hydroxycinnamyl alcohols, p-coumaryl, coniferyl, and sinapyl alcohols, which contribute to lignin biosynthesis, and these three hydroxycinnamyl alcohols give rise to the p-hydroxyphenyl (H), guaiacyl (G), and syringyl (S) units of the lignin polymer, respectively [17]. And O-Methyltransferases (OMTs) play an important role in regulating these secondary metabolic processes involved in lignin biosynthesis. OMTs are generally classified into two types, including caffeic acid O-methyltransferase (COMT) and caffeoyl-coenzyme A O-methyltransferase (CCoAOMT), of which COMT controls the S unit pathway and CCoAOMT affects the S and G unit pathways[18]. COMT can catalyse the O-methylation at the 5 position of the aromatic ring, and CCoAOMT functions to form the 3 position of the aromatic ring [19, 20]. The hydroxylation and methylation steps are crucial to determine the lignin composition and the S/G ratio is a major determinant of lignin quality [18].

In light of its significance, the CCoAOMT gene family has been systematically investigated and analysed in many plants, such as Arabidopsis and rice [21], citrus [22], switchgrass [23], dove tree [24], tea plant [25] and sorghum [26]. In wheat, Nguyen et al. analysed the expression patterns and potential functions of some genes involved in lignin biosynthesis including several CCoAOMTs, and indicated that lodging resistance, tolerance against biotic and abiotic stresses and feedstock quality of wheat biomass were closely associated with its lignin content [27]. Ma and Luo verified that *TaCCoAOMT*1 was an important gene for regulating lignin biosynthesis, which is critical for stem development [28].

Although some CCoAOMT genes have been functionally characterized in wheat, the genomic organization, evolutionary relationship and regulatory module of the wheat CCoAOMT family are not well understood at present, especially in association with FHB. In this study, we performed an *in silico* genome-wide search method to identify and characterize the CCoAOMT family in wheat using updated reference genome information. Then, their phylogenetic relationships, conserved motifs and cis-elements were systematically analysed. Furthermore, the expression patterns and co-expression network of TaCCoAOMT genes under *Fg* treatment were studied and FHB-responsive TaCCoAOMTs as well as the regulatory modules were obtained. Finally, the genetic diversity and divergence of CCoAOMT genes in different *Triticum* species were also investigated based on resequencing data to reveal the evolutionary effect of this family during wheat formation.

## Results

### Genome-wide identification of CCoAOMT genes in wheat

Using the genome-wide search method described in the Methods section, a total of 21 CCoAOMT genes were detected in the wheat genome. Since there is no standard nomenclature, these identified CCoAOMT genes were named as *TaCCoAOMT1* to *TaCCoAOMT21* based on their chromosome location (Table 1). These *TaCCoAOMT* genes were mainly located on group 7 chromosomes (66.67 %), but not on groups 2, 3 and 6 chromosomes. The sizes of *TaCCoAOMT* genes ranged from 447 (*TaCCoAOMT17*) to 4567 (*TaCCoAOMT12*) bp in length. The average lengths of the CDS and amino acid sequences were 761 bp and 253 aa, respectively. The isoelectric point (pI) ranged from 4.89 (*TaCCoAOMT15*) to 11.11 (*TaCCoAOMT17*) and the molecular weight (Mw) ranged from 16284.2 (*TaCCoAOMT17*) to 34158.48 (*TaCCoAOMT21*), respectively. A search for orthologues of *TaCCoAOMT* genes revealed that 20 (95.24 %) of the *TaCCoAOMTs* had orthologues in Arabidopsis and rice, with the only exception being *TaCCoAOMT6*. Subcellular localization prediction showed that most of them were located in the cytoplasm and chloroplasts, and only one gene was located in the mitochondria and nucleus, respectively. In these 21 *TaCCoAOMT* genes, we also found four homoeologous gene groups with each containing A, B and D homoeologous copies, and all of them were localized on chromosome group 7, resulting in chromosome group 7 having the most abundant *TaCCoAOMT* genes.

**Table 1:** Characteristics of the CCoAOMT genes identified in wheat

TaCCoAOMT	Transcript	Gene Length (bp)	Protein Length (aa)	Exon number	Splice variant	pI	Mw (Da)	Subcellular localization	Orthologs
TaCCoAOMT1	TraesCS1B02G023200.2	2088	238	5	2	5.03	26054.06	Cytoplasmic	AtCCoAMT3
TaCCoAOMT2	TraesCS1B02G049800.1	1168	210	2	1	5.55	23350.86	Cytoplasmic	AtCCoAMT3
TaCCoAOMT3	TraesCS1D02G019000.1	1245	248	2	1	5.02	27035.03	Cytoplasmic	-
TaCCoAOMT4	TraesCS4A02G442400.1	1355	246	2	1	4.98	27050.77	Cytoplasmic	OsCCoAOMT3
TaCCoAOMT5	TraesCS4D02G362500.1	942	190	1	1	5.04	20387.36	Chloroplast	OsCCoAOMT5
TaCCoAOMT6	TraesCS5A02G257600.1	1136	252	5	2	5.39	27304.38	Chloroplast	-
TaCCoAOMT7	TraesCS5D02G265900.1	1586	242	5	1	5.15	26189.03	Chloroplast	OsCCoAOMT6
TaCCoAOMT8	TraesCS7A02G068600.1	1648	288	4	2	7.01	31886.32	Mitochondrial	OsCCoAOMT5
TaCCoAOMT9	TraesCS7A02G127600.1	1093	284	3	1	5.04	31491.93	Cytoplasmic	OsCCoAOMT1
TaCCoAOMT10	TraesCS7A02G240300.1	1753	247	2	1	4.99	27088.92	Cytoplasmic	OsCCoAOMT3
TaCCoAOMT11	TraesCS7A02G240400.1	1219	245	3	1	4.95	27071.88	Cytoplasmic	OsCCoAOMT4
TaCCoAOMT12	TraesCS7A02G302500.1	4567	300	10	2	8.59	32885.04	Chloroplast	AtCCoAOMT5
TaCCoAOMT13	TraesCS7B02G027200.1	1729	267	4	1	5.11	29506.65	Cytoplasmic	OsCCoAOMT1
TaCCoAOMT14	TraesCS7B02G135900.1	1854	302	2	1	5.54	33139.88	Chloroplast	OsCCoAOMT3
TaCCoAOMT15	TraesCS7B02G136000.1	1734	245	3	1	4.89	27124.85	Cytoplasmic	OsCCoAOMT4
TaCCoAOMT16	TraesCS7B02G202600.1	4218	294	9	1	7.57	32151.1	Chloroplast	OsCCoAOMT2
TaCCoAOMT17	TraesCS7D02G063100.1	447	148	1	1	11.11	16284.2	Nuclear	AtCCoAOMT3
TaCCoAOMT18	TraesCS7D02G126000.1	2465	260	4	1	5.11	28864.96	Cytoplasmic	OsCCoAOMT1
TaCCoAOMT19	TraesCS7D02G239200.1	1548	247	2	1	4.98	27074.9	Cytoplasmic	OsCCoAOMT3
TaCCoAOMT20	TraesCS7D02G239400.1	1185	245	3	1	4.94	27121.94	Cytoplasmic	OsCCoAOMT3
TaCCoAOMT21	TraesCS7D02G297800.1	3828	311	9	2	8.38	34158.48	Chloroplast	OsCCoAOMT2

### Phylogenetic relationship, exon-intron structure and conserved motif analysis

**Fig. 1:**
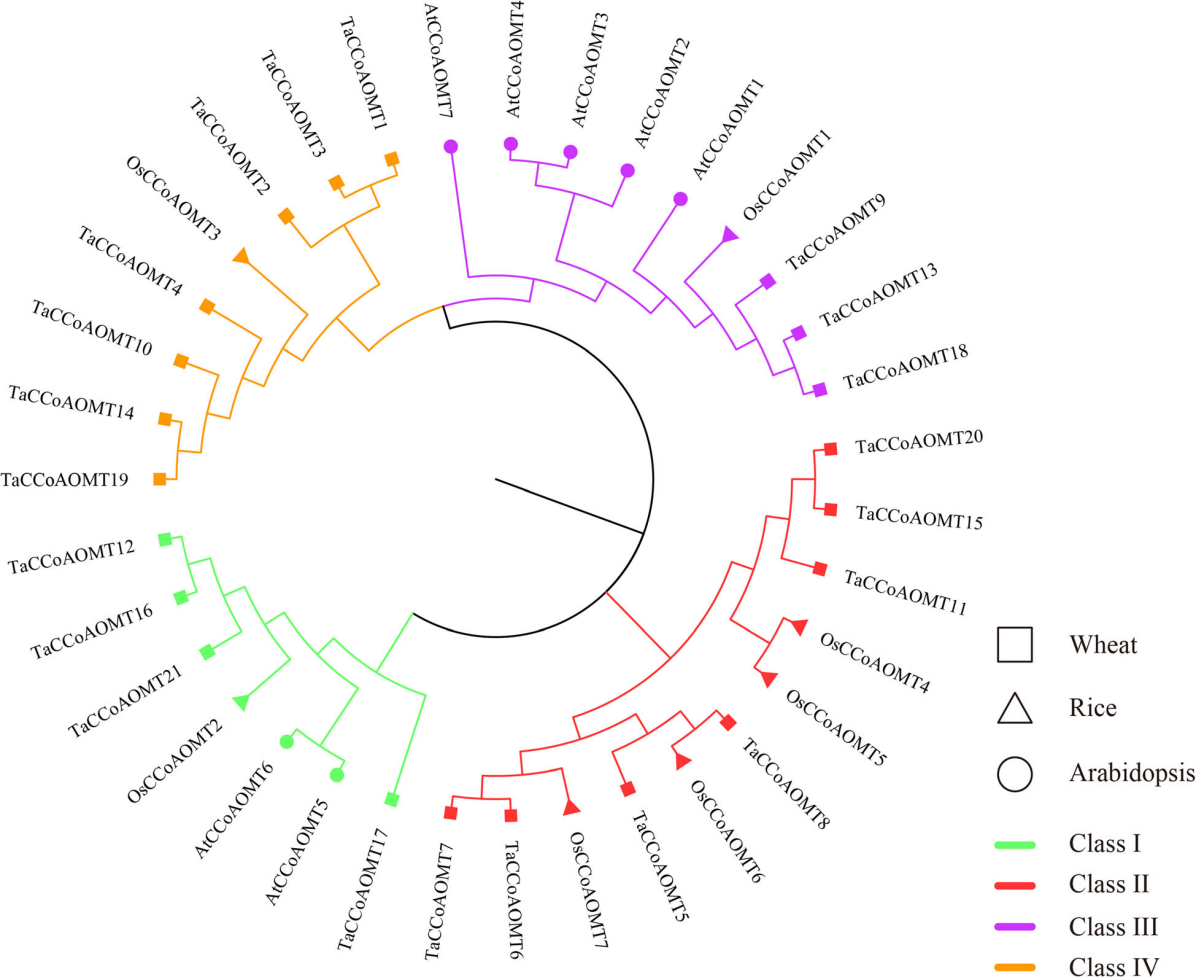
Phylogenetic relationship of CCoAOMT genes in Arabidopsis, rice and wheat

A phylogenetic tree was constructed using the full-length protein sequences of the CCoAOMT genes in wheat, Arabidopsis and rice (Fig. 1). The corresponding nomenclature information of CCoAOMT genes in Arabidopsis and rice is provided in Table S1. The results indicated 4 groups (designated classes I to IV) with 4, 7, 3 and 7 *TaCCoAOMT* genes, respectively. Meanwhile, *TaCCoAOMT* and *OsCCoAOMT* genes were distributed in all of groups, but AtCCoAOMT genes were mainly clustered in class III, and there were just two genes (*AtCCoAOMT5* and *AtCCoAOMT6*) in class I.

**Fig. 2:**
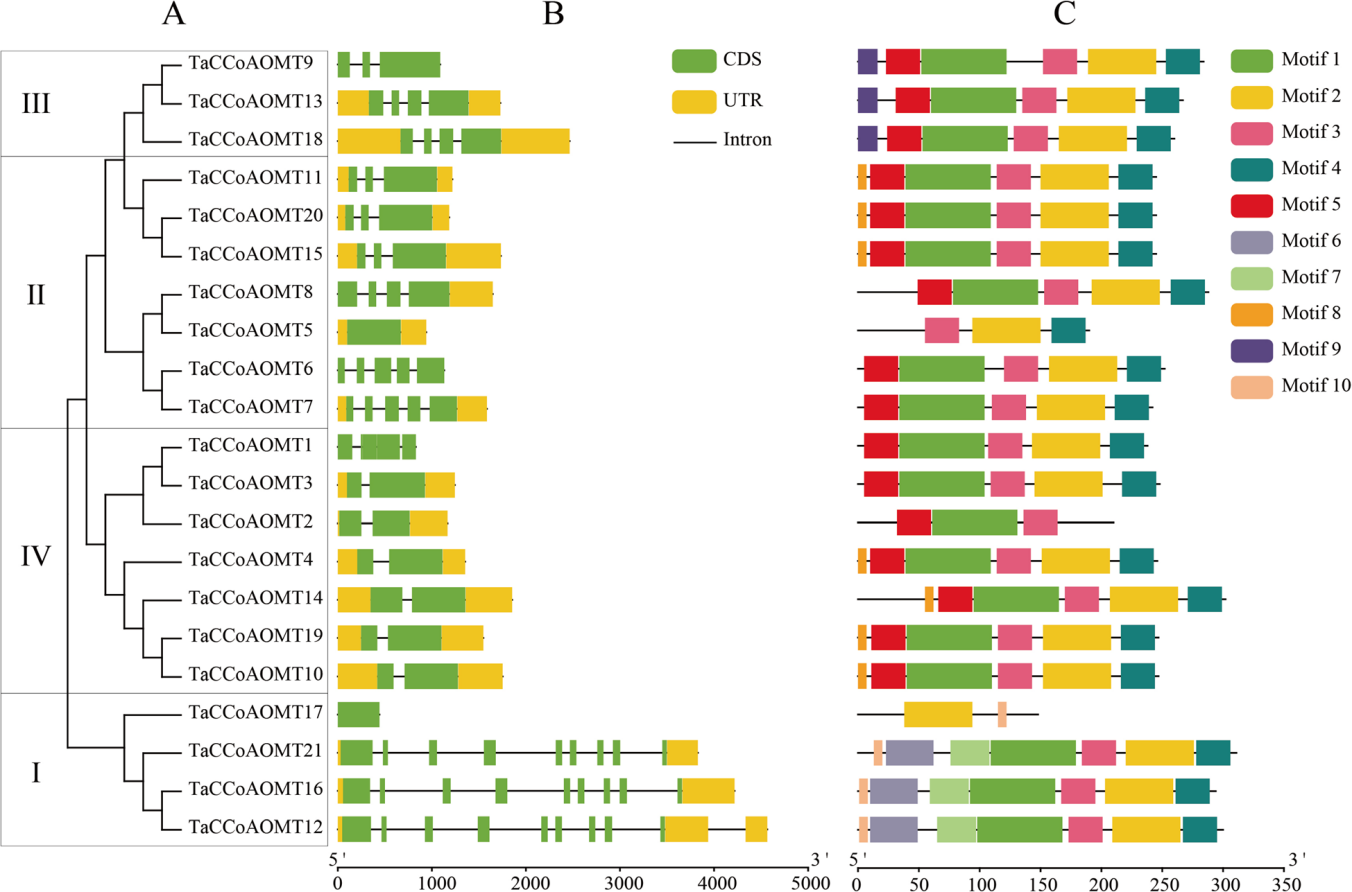
Phylogenetic tree (**A**), gene structure (**B**) and conserved motif analysis (**C**) of the CCoAOMT gene family in wheat

Then, the exon-intron and motif structures of *TaCCoAOMTs* were further analysed (Fig. 2). The exon number of *TaCCoAOMTs* ranged from 1 to 10, of which two genes contained only one exon, and 85.71 % of genes had 5 exons or less (Fig. 2B). Furthermore, 10 high confidence motifs were predicted (Fig. 2 C and Figure S1). Compared to other classes, motifs 6 and 9 were exclusively found in classes I and III, respectively. Although few differences in motifs were found between classes II and III, the exon number of *TaCCoAOMT* genes in class II was greater that in class III. Motif 2 was identified in 20 (95.24 %) *TaCCoAOMTs*, apart from *TaCCoAOMT2* and motif 3 was also identified in 20 *TaCCoAOMTs* apart from *TaCCoAOMT17*. Motif 1, 2, 3, 4 were abundant in *TaCCoAOMTs* (Fig. 2), and all of them were found to be related to O-methyltransferase based on PFAM analysis, which further supported the prediction. Meanwhile, the members in the same groups shared the similar exon-intron structures and motif compositions (Fig. 2).

### Cis-element analysis of TaCCoAOMTs

A total of 44 types of cis-elements were identified in the 1.5-kb genomic sequences upstream from the transcription start sites (TSS) of *TaCCoAOMT* genes, with functions primarily associated with the stress response (Table S2 and Figure S2). The CAAT-box was identified in all *TaCCoAOMTs* (21), followed by the CGTCA-motif (19) and TGACG-motif (19). Together with the CGTCA motif and TGACG motif, the ABRE motif related to abscisic acid responsiveness was identified in 18 *TaCCoAOMT*s. In addition, CCAAT-box (MYBHv1 binding site), MBS (MYB binding site involved in drought-inducibility), TCA-element (cis-acting element involved in salicylic acid responsiveness), TC-rich repeats (cis-acting element involved in defence and stress responsiveness), MRE- (MYB binding site involved in light responsiveness), SARE- (cis-acting element involved in salicylic acid responsiveness) and WUN-motif (wound-responsive element) were also identified in 13, 7, 7, 2, 1, 1 and 1 *TaCCoAOMT*s, respectively.

To obtain some clues about the biological function of *TaCCoAOMTs*, we performed GO (gene ontology) enrichment analysis of them with all wheat proteins as background. The results showed that *TaCCoAOMTs* were significantly enriched in 10 terms in biological process, 10 terms in molecular function and one in cell component (Figure S3 and Table S3). Among them, lignin biosynthetic process (GO:0009809), O-methyltransferase activity (GO:0008171) and caffeoyl-CoA O-methyltransferase activity (GO:0042409) were significantly enriched, verifying the correlation between *TaCCoAOMTs* and lignin biosynthesis.

### Expression patterns of ***TaCCoAOMT*** genes in different tissues and under abiotic stresses

**Fig. 3:**
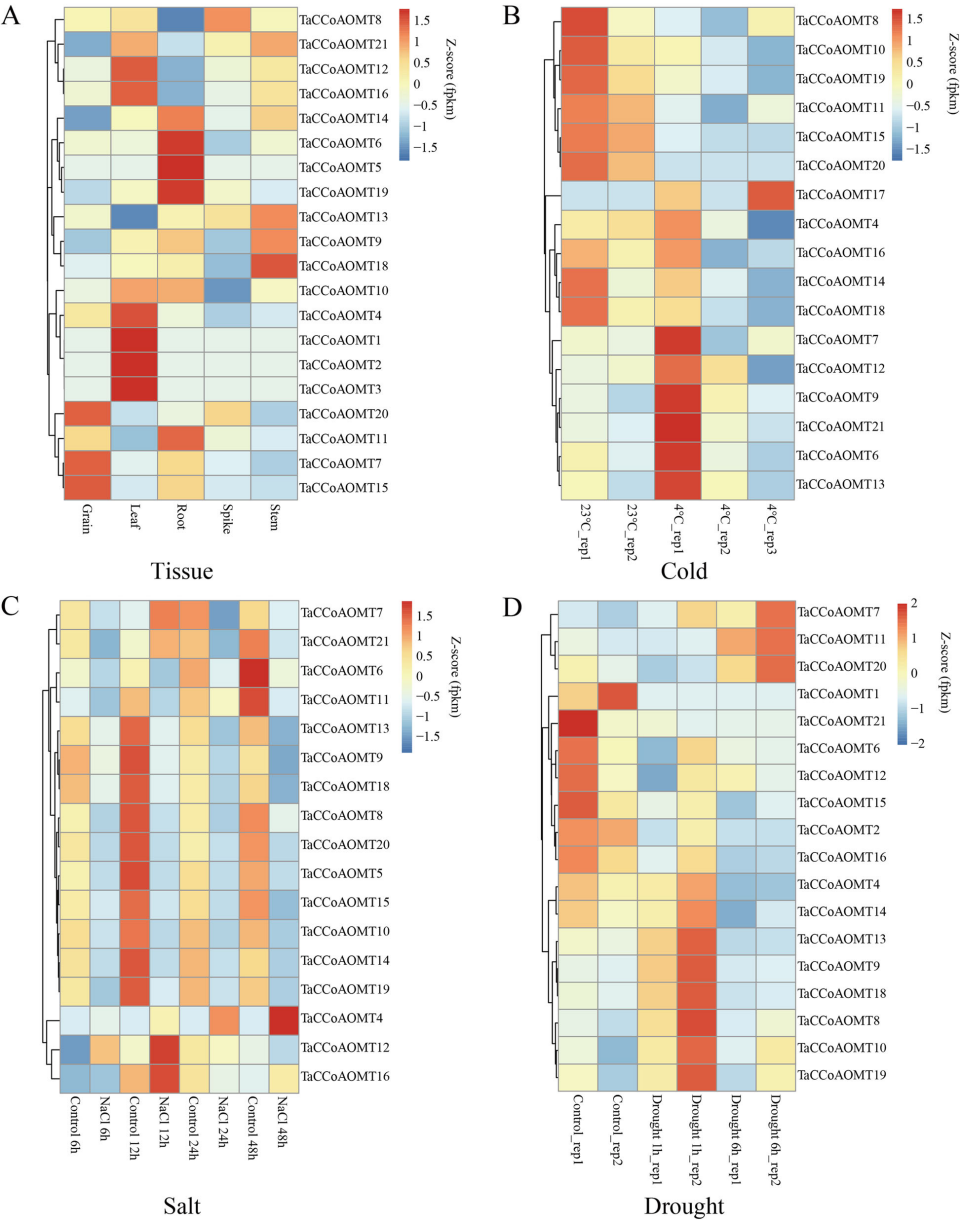
Expression patterns of *TaCCoAOMT* genes in different tissues (**A**) and under diverse abiotic stresses (**B** to **D**)

The spatio-temporal expression patterns of *TaCCoAOMTs* were investigated using RNA-seq samples of different tissues as well as under low temperature, salt, heat and drought stresses. A total of 20 *TaCCoAOMTs* were found to be expressed in different tissues and some genes exhibited tissue-specific expression. Totally, 3 genes (*TaCCoAOMT7, 15, 20*) were highly expressed in grain (Fig. 3 A). Meanwhile, 7 and 6 genes displayed high expression in leaves and roots, respectively. *TaCCoAOMT8* was expressed only in the spike while 3 genes (*TaCCoAOMT9, 13, 18*) showed specifically high expression in stem tissue.

Under low temperature treatment (Fig. 3B and Table S4), we found 3 differentially expressed *TaCCoAOMTs*, of which *TaCCoAOMT9* displayed up-regulated expression, while *TaCCoAOMT20* and *TaCCoAOMT15* were down-regulated. Under salt stress (Fig. 3 C and Table S4), 3 genes (*TaCCoAOMT5, 8, 20*) were significantly down-regulated and two (*TaCCoAOMT4, 12*) were up-regulated at 6 h, and 4 and 5 *TaCCoAOMTs* were significantly down-regulated and up-regulated at 12 h respectively. Only *TaCCoAOMT4* and *TaCCoAOMT5* showed differential expression at 24 and 48 h, respectively. Under drought stress (Fig. 3D and Table S4), 5 and 6 *TaCCoAOMT*s were differentially expressed at 1 and 6 h, respectively, of which 3 genes (*TaCCoAOMT2, 13, 20*) were differentially expressed at both time points.

### Comprehensive analysis of the expression profiles of ***TaCCoAOMT*** genes under ***Fg*** infection

**Fig. 4:**
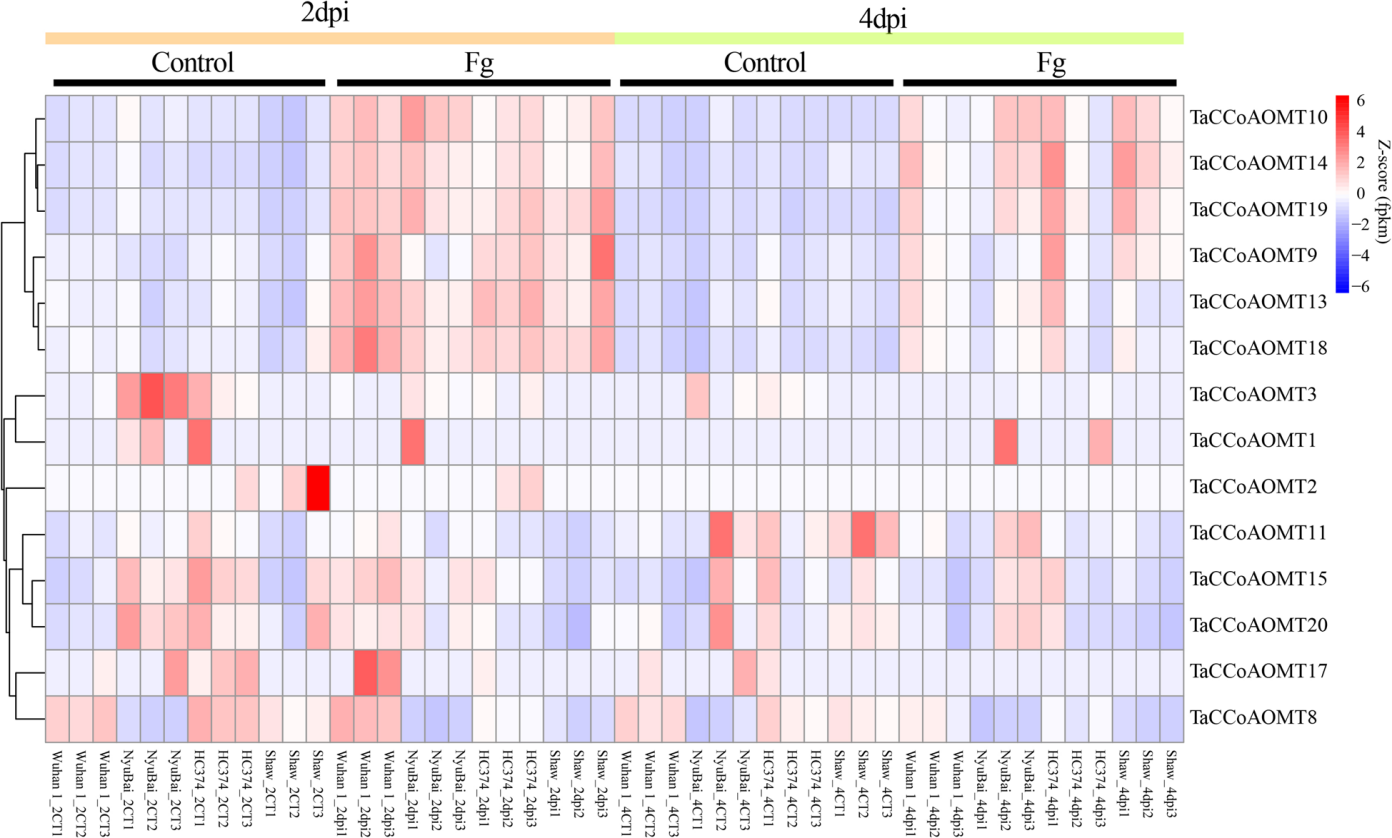
Expression patterns of ***TaCCoAOMT*** genes in four different wheat genotypes inoculated with water (Mock) or ***Fg*** at 2 dpi and 4 dpi. Wuhan 1, HC374, Nyubai and Shaw are the names of the four wheat varieties. dpi: days post inoculation

According to a previous study, lignin biosynthesis process-related genes played a significant role in the response to *Fg* infection [29]. To identify the *TaCCoAOMTs* involved in the *Fg* response, we further used 48 RNA-seq samples from *Fg* infection and mock samples of three FHB-tolerant varieties (HC374, NyuBai, Wuhan 1) and one FHB-susceptible variety (Shaw) at 2 days and 4 days post inoculation (dpi) [30]. Expression patterns of these *TaCCoAOMT*s showed obvious differentiation between 2 and 4 dpi. At 2 dpi, 13, 12, 12 and 11 of *TaCCoAOMTs* were expressed in genotypes HC374, NyuBai, Wuhan 1 and Shaw, respectively (Fig. 4 and Table S5), of which down-regulated genes accounted for 64.3 % and up-regulated genes accounted for 35.7 %. The number of DEG in Wuhan1 was the lowest, and that in Shaw was the highest. Furthermore, three homoeologues of *TaCCoAOMT (8, 15*, 20*)* showed differential expression among three varieties (HC374, NyuBai, Shaw), of which *TaCCoAOMT 8* was differentially expressed among all four varieties. At 4 dpi, there were 12, 11, 10 and 10 *TaCCoAOMT* genes were expressed in genotypes HC374, NyuBai, Wuhan 1 and Shaw (down-regulated: 58.3 %; up-regulated: 41.7 %), respectively (Fig. 4 and Table S5), of which *TaCCoAOMT 11* showed differential expression among all four varieties. Genotype Shaw also had the largest number of DEGs, while the number of DEGs in the other three varieties was basically the same. In general, the number of down-regulated genes was greater than the number of up-regulated genes after *Fg* injection, and the number of DEGs in Shaw was the hihgest. Compared to different dpi, the number of DEGs in Wuhan 1 increased from 2 dpi to 4 dpi, while the number of DEGs in the other three varieties decreased (HC374, NyuBai) or remained unchanged (Shaw). By comparing resistant and susceptible varieties, *TaCCoAOMT3* and *TaCCoAOMT17* showed significant down-regulated expression only in three resistant varieties; at the same time, *TaCCoAOMT2* was significantly down-regulated only in the susceptible variety. It is interesting that *TaCCoAOMT 10, 14* and *19* still showed up-regulated expression between Fg infection and mock samples in all four varieties, suggesting that they might play a vital role in the response to *Fg* infection in wheat.

**Fig. 5:**
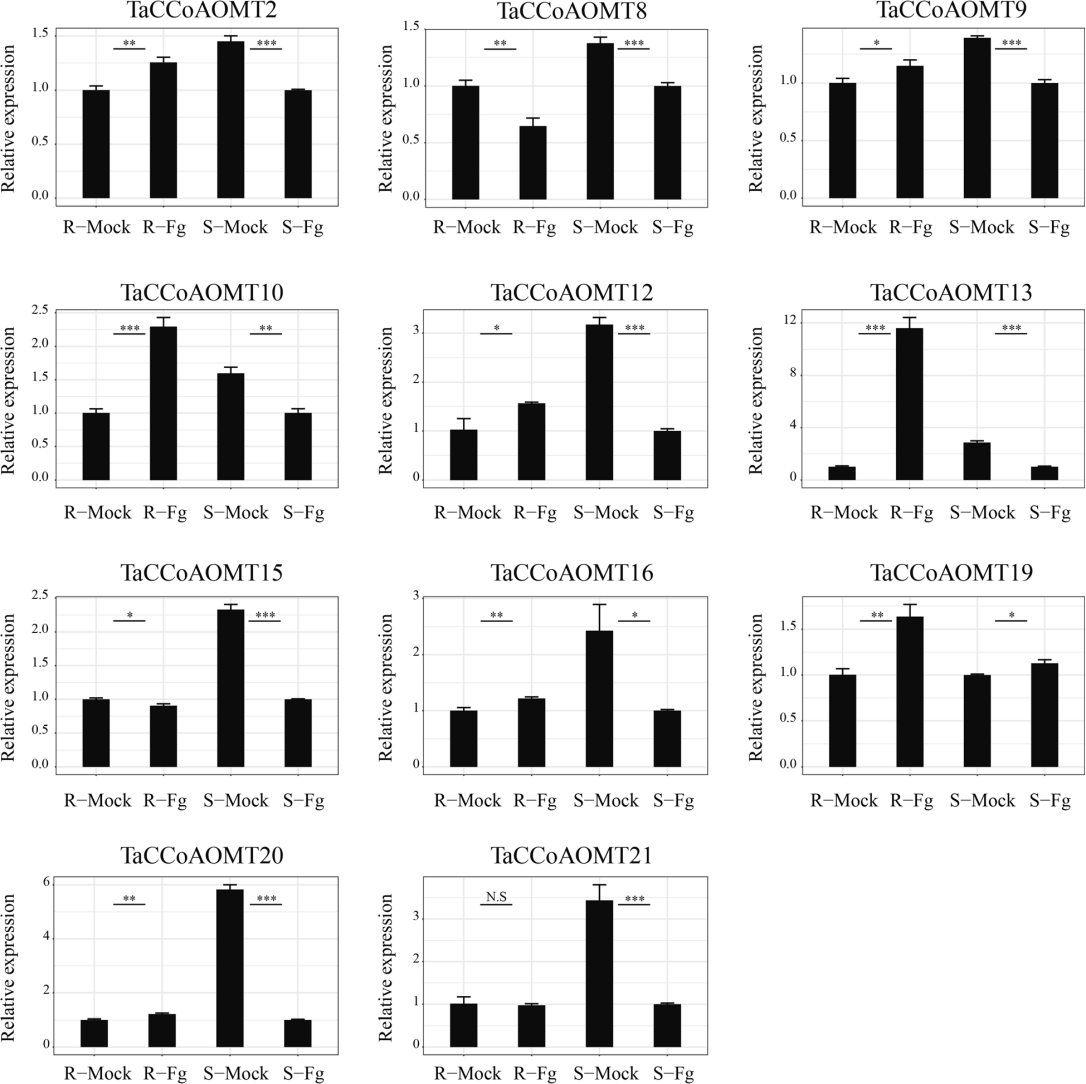
qRT-PCR analysis of 11 FHB-responsive ***TaCCoAOMT*** genes. R-Mock: resistant genotype R75 under control condition; R-Fg: resistant genotype R75 under *Fg* treatment; S-Mock: susceptible genotype S98 under control condition; S-Fg: susceptible genotype under *Fg* treatment. Significance between mock and *Fg* infection samples in resistant genotype R75 and susceptible genotype S98 were analyzed using student’s t-test (*P < 0.05, **P < 0.01, ***P < 0.001, N.S: Not significant). The exact sample size for each experimental condition of each *TaCCoAOMT* gene was 15 spikes, classified into three biological replicates. The bars display the means of *TaCCoAOMT* gene expression in the mock or control. The error bars represent standard error of mean (SEM) of the three separate technical replicates of qRT-PCR experiments

To further verify the expression of *TaCCoAOMT*s under *Fg* infection, 11 genes were randomly selected to validate their expression between the resistant and susceptible recombinant inbred lines by qRT-PCR analysis (Fig. 5). The results demonstrated that the expression trend of these genes was consistent with that of RNA-seq analysis. Compared to mock, almost all of the tested genes displayed differential expression under *Fg* infection in both resistant and susceptible genotypes, of which *TaCCoAOMT19* were up-regulated after *Fg* infection in both resistant and susceptible genotypes, while *TaCCoAOMT8, 15, 21* showed down-regulated expression in both genotypes. It is obvious that the expression levels of 7 genes (*TaCCoAOMT2, 9, 10, 12, 13, 16, 20*) were up-regulated in the resistant genotype but down-regulated in the susceptible genotype, suggesting that they might be involved in *Fg* resistance.

### Co-expression network and regulation module of FHB-responsive TaCCoAOMTs

**Fig. 6:**
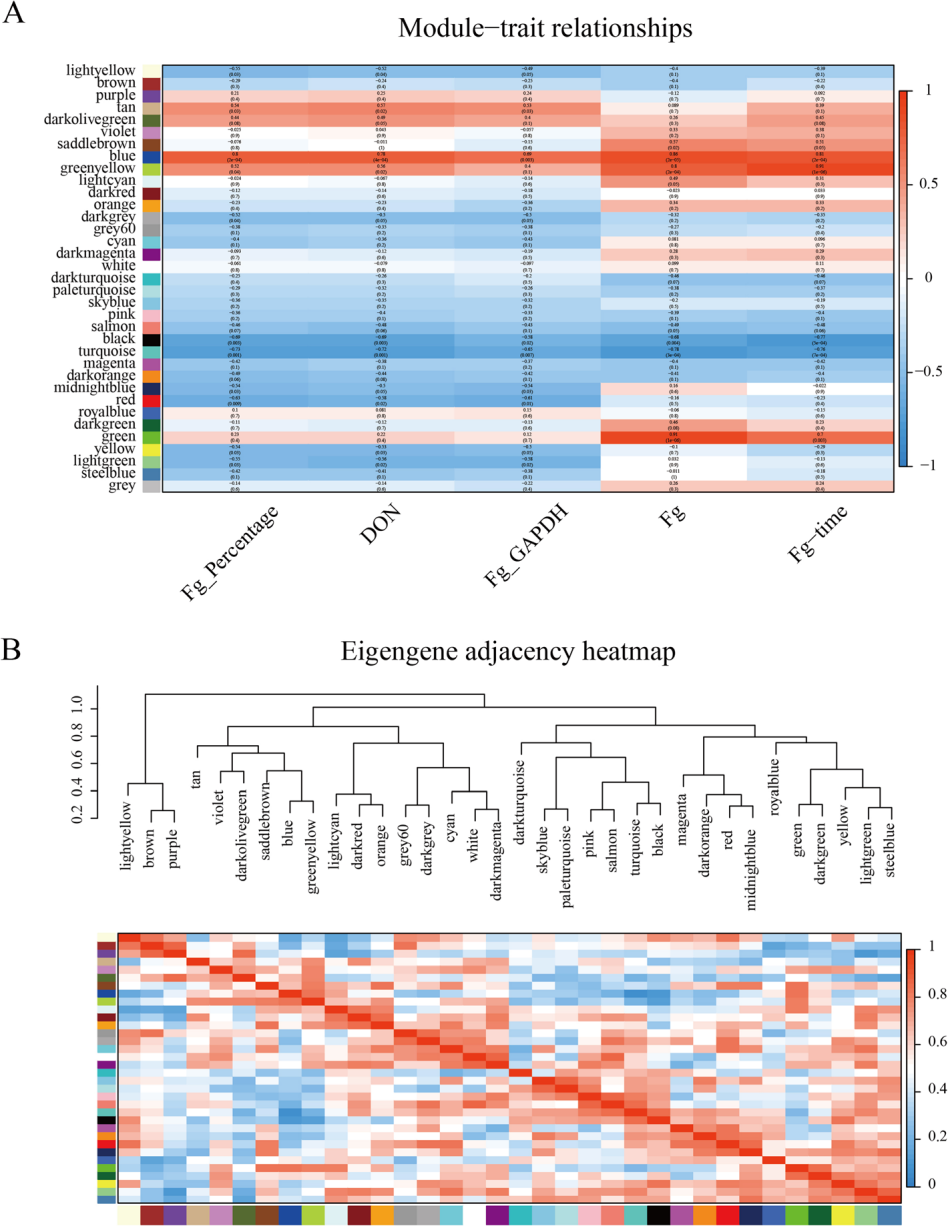
WGCNA based on 48 RNA-seq samples in wheat. (**A**). Correlation between co-expression modules and traits. A negative value represents the negative correlation, and a positive value represents the positive correlation. (**B**). Heatmap of correlation between co-expression modules

**Fig. 7:**
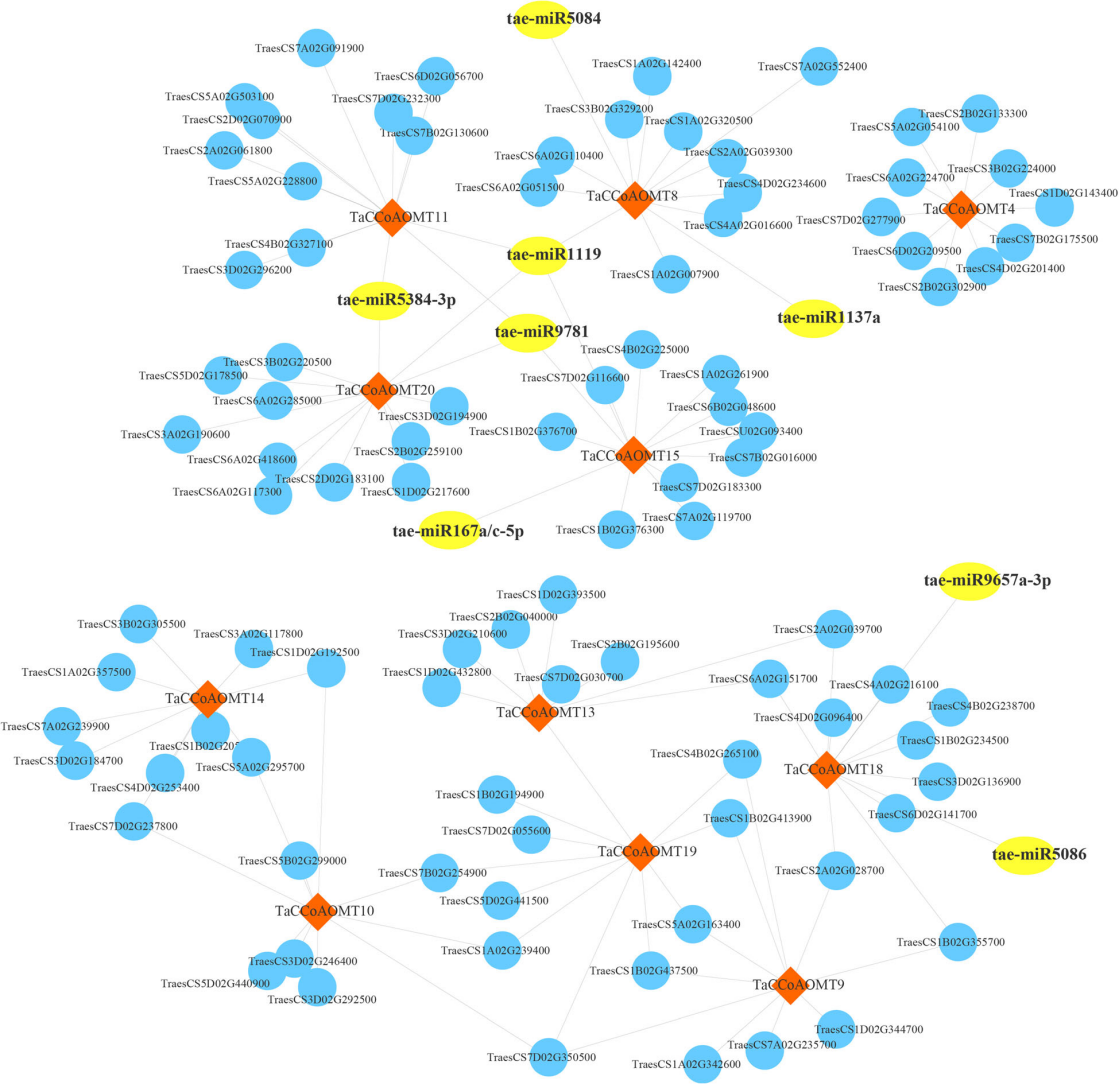
miRNA-mRNA interaction network of *TaCCoAOMT* genes under *Fg* stress

To better understand the function and regulatory network of the identified FHB-responsive *TaCCoAOMTs*, we further constructed a WGCNA co-expression network based on these RNA-seq data. By constructing a weighted correlation network, 34 co-expression modules were obtained (Fig. 6). Then, we linked the co-expression modules with the available phenotypic data of the *Fg* inoculation, including percentage (Fusarium oxysporum inoculum), DON (deoxynivalenol), GAPDH (glyceraldehyde-3-phosphate dehydrogenase content) and inoculation time [30]. The results showed that tan, darkolivegreen, blue and greenyellow modules were highly correlated with *Fg* percentage, DON, GAPDH and inoculation time, respectively (Fig. 6 A). Interestingly, 2, 5, 1, 1 and 2 FHB-responsive *TaCCoAOMTs* were found in the blue, green, light yellow, turquoise and yellow modules, respectively. Furthermore, these modules were also associated with *Fg* inoculation (Fig. 6B). A total of 93 genes in these modules showed similar expression patterns. GO enrichment analysis found that they were mainly enriched in terms related to defence response, such as GO:0052544 (defense response by callose deposition in cell wall), GO:0010294 (abscisic acid glucosyltransferase activity) and GO:0000165 (MAPK cascade). It is obvious that the *TaCCOAOMTs* are located at the hubs of these modules. Among them, *TaCCoAOMT19*, which is associated with FHB tolerance, could interact with *TaCCoAOMT13* to regulate 19 other genes to form a regulatory module (Fig. 7), which might play a potentially important role in FHB tolerance.

Furthermore, we predicted the miRNAs that could target on *TaCCoAOMTs* to regulate their expression. Totally, 15 *TaCCoAOMT* genes were found to be targeted by 25 miRNAs (Table S6) and 50 miRNA-TaCCoAOMT interactions were constructed. Combined with the miRNA-TaCCoAOMT relationship and co-regulation modules of *TaCCoAOMTs*, we further obtained the miRNA-mediated networks associated with the FHB response and resistance involving TaCCoAOMTs in wheat (Fig. 7), which provided some insights into the regulation of *TaCCoAOMTs* expression to control lignin biosynthesis and enhanced FHB tolerance through a post-transcriptional approach.

### Genetic diversity and haplotype analysis of the CCoAOMT family in wheat and its relatives

**Fig. 8:**
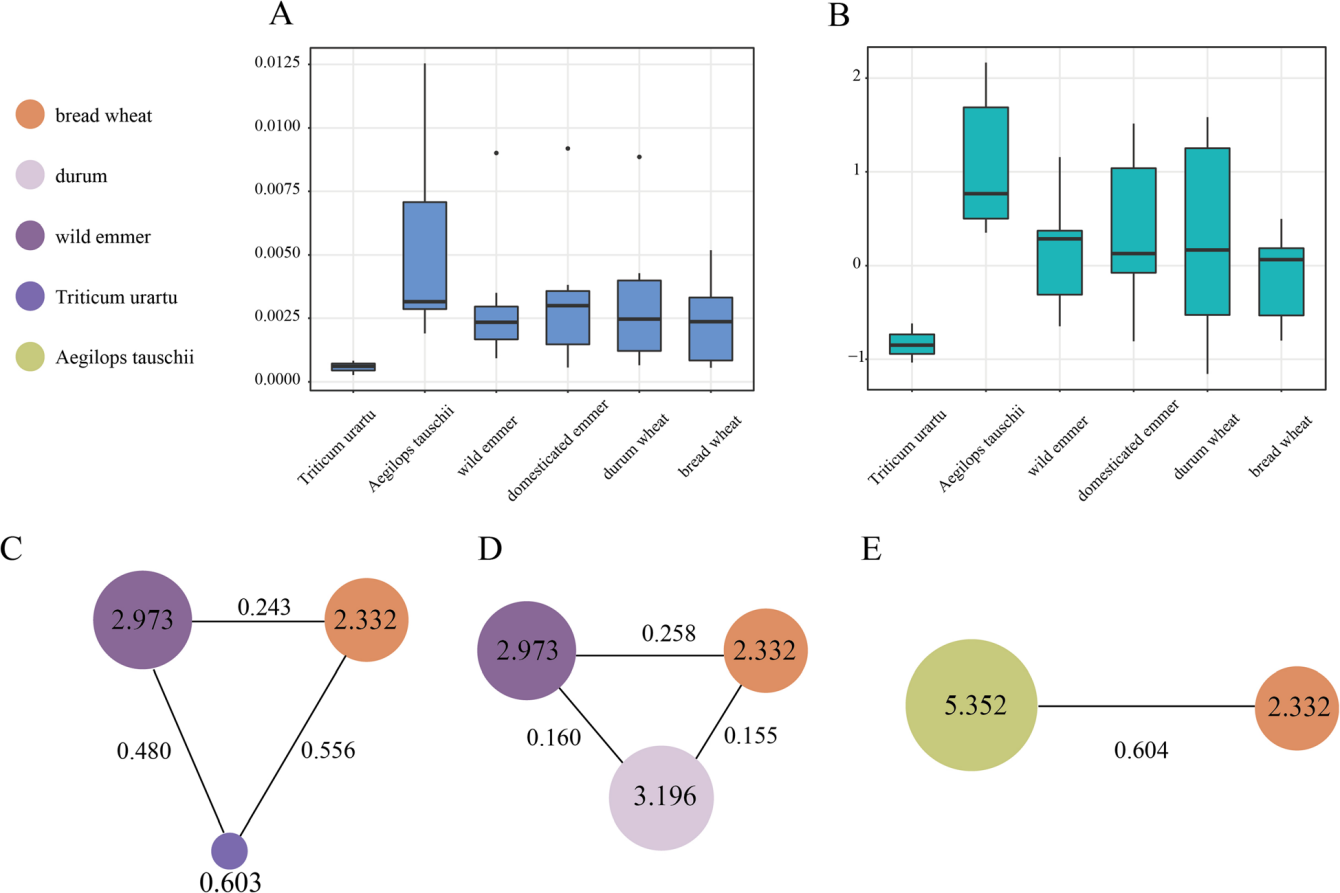
Genetic diversity, population divergence and Tajima’s D across the ***TaCCoAOMT*** genes in bread wheat and its relative populations. (**A**). Nucleotide diversity across five Triticum species among the whole genome. (**B**). Tajima’s D across five Triticum species in the whole genome. (**C**). Nucleotide diversity (π × 10^− 3^) and population divergence (Fst) across the *Triticum urartu*, wild emmer and bread wheat among the A genome. (**D**). Nucleotide diversity and population divergence across the wild emmer, durum and bread wheat among the B genome. (**E**). Nucleotide diversity and population divergence across the *Aegilops tauschii* and bread wheat among the D genome

Based on the resequencing data of Triticum species [31], the genetic variations of CCoAOMT genes in wheat and its diploid and tetraploid relatives were investigated, including the nucleotide diversity (π), population divergence (Fst) and Tajima’s D index. The average values of π in *Triticum urartu*, *Aegilops tauschii*, wild emmer, domesticated emmer, durum wheat and bread wheat were 0.000565, 0.00535, 0.00297, 0.00321, 0.00320 and 0.00233, respectively (Fig. 8 A), and the average values of Tajima’s D was − 0.834, 1.094, 0.145,0.370, 0.292 and − 0.137, respectively (Fig. 8B). Due to few SNPs identified from the re-sequencing data, *T. urartu* showed the abnormally lower value of nucleotide diversity. *Ae. tauschii* displayed the highest nucleotide diversity in CCoAOMT genes, while bread wheat had the lowest nucleotide diversity apart from *T*. *urartu*, with a value that decreased by 2 times, suggesting that a significant genetic bottleneck occurred in the CCoAOMT gene family during wheat evolution. Then, the gene flow and genetic divergence between wheat subgenome and its relatives were also detected. In the A subgenome, the *Fst* value between bread wheat and *T. urartu* was 0.556, ranking as the largest, followed by that of wild emmer and *T. urartu* with the value of 0.480, and bread wheat and wild emmer with a value of 0.248, indicating the high divergence between bread wheat and *T. urartu*, compared to that of wild emmer at the A subgenome level from the perspective of CCoAOMT gene family (Fig. 8 C). In the B subgenome, the divergence between bread wheat and wild emmer was larger than that of durum and wild emmer, and bread wheat was closer to durum wheat than wild emmer wheat (Fig. 8D). In the D subgenome, the Fst value of between bread wheat and *Ae. tauschii* was 0.604 (Fig. 8E). Overall, the genetic divergence at the D subgenome was highest, followed by the A and B subgenomes.

Finally, we identified the haplotype organization and frequency of each *TaCCoAOMT* gene in these populations based on the resequencing data (Table S7). A total of 13 *TaCCoAOMT* genes were found to have the genetic variations among these populations, of which 4, 2 and 7 genes were located in the A, B, D subgenomes, respectively. Then, the main haplotype and its frequencies were investigated (Figure S4, S5 and S6). It is obvious that the percentage of the main haplotype in cultivated wheat was significantly larger than that of wild species at all subgenome levels, indicating that the artificial selection exerted on these CCoAOMT genes resulted in the decline of genetic diversity and genetic bottlenecks during wheat domestication and improvement processes.

## Discussion

Lignin is the main component of the cell wall and is involved in the response to abiotic and biotic stresses [19]. The characteristics of the CCoAOMT gene involved in the lignin synthesis pathway has been analysed in Arabidopsis, sorghum, and other plants. In this study, we identified 21 CCoAOMTs in wheat at the genome level. Based on the phylogenetic relationship, these *TaCCoAOMTs* were classified into four groups, and the *TaCCoAOMTs* belonging to the same group shared similar gene structures and motif organizations. In sorghum, the CCoAOMT proteins were classified into clade 1a, clade 1b, clade 1c and clade 2, of which clade 1a was the orthologous gene with *AtCCoAOMT1* and *OsCCoAOMT1*, which was considered as the true CCoAOMT gene, while the other classes were considered as CCoAOMT-like genes [26]. In our results, *AtCCoAOMT1* and *OsCCoAOMT1* were clustered into the same group (class III), which was consistent with that of sorghum. Meanwhile, CCoAOMT genes in rice and wheat can be found in each group, but *AtCCoAOMTs* just were found in classes I and III, indicating that there was some divergence between the monocot and dicot CCoAOMT families. Additionally, classes I and III have the specific motifs 6 and 9, respectively. Although no specific motif was found in classes II and IV, their gene structures displayed differences with the obvious variations in exon number, suggesting that more types of splice variants or binding-sites might be present in these two classes.

Based on RNA-seq samples, the expression levels of these wheat CCoAOMT genes in different tissues were comprehensively analysed, and the tissue-specific *TaCCoAOMT*s were obtained, which provided useful targets for further functional study. Simultaneously, 7 *TaCCoAOMTs* showed significantly up-regulated expression after *Fg* treatment compared to mock treatment, of which 3 genes (*TaCCoAOMT10, 14* and *19*) were shared by three resistant varieties and one susceptible variety, suggesting that they might play an important role in the response to FHB. Interestingly, *TaCCoAOMT10, 14* and *19* were the A, B and D homoeologous copies of the same homoeologous group respectively, of which the expression level of *TaCCoAOMT19* was the highest, indicating its dominant role in the response to FHB and asymmetric expression between homoeologous copies. Otherwise, the difference between the four varieties showed the potential FHB tolerance candidates. For example, *TaCCoAOMT3* and *17* were specifically down-regulated in three resistant varieties, indicating the repression effect of them on Fg tolerance. *TaCCoAOMT2* displayed the specifical down-regulation in susceptible variety Shaw, which further demonstrates the uniqueness of the sensitive variety.

It is well known that cis-regulatory elements can regulate gene expression levels by binding to corresponding transcription factors, and then might determine the specific expression patterns in different tissues and stresses [32]. The CGTCA-motif and TGACG-motif, which are related to MeJA-responsiveness, were identified in the promoter regions of all up-regulated *TaCCoAOMTs*. A previous study proved that MeJA could not only help to delay the necrosis process of susceptible varieties in wheat but also increase the activities of enzymes related to pathogen defence [33]. Therefore, these up-regulated *TaCCoAOMTs* containing CGTCA-motif and TGACG-motif might play a crucial role in regulating FHB tolerance through MeJA mediation. We further reconstructed the co-expression network by the WGCNA method based on 48 RNA-seq samples of four wheat varieties [30]. Results showed that seven modules were significantly associated with *Fg* infection, of which 5 modules harboured the *TaCCoAOMT* genes as the hub factors, and two modules (blue and green) were positively correlated with *Fg* infection and three modules (lightyellow, turquoise and yellow) were negative. These modules provided some insight into the genetic basis and regulatory network of *TaCCoAOMT*, which might contribute to the molecular mechanism underlying FHB resistance. Furthermore, eight microRNAs were found to target five *TaCCoAOMT* genes involved in the *Fg* infection-related modules, including tae-miR167a and tae-miR1119. Previous studies have been reported that miR167a could meditate auxin signalling to respond to biotic stresses in tomato [34], and miR1119 was proven to regulate the expression of actin under stress conditions and activate the plant defence signalling pathway in barley [35]. We postulated that these two miRNAs also play the regulatory role in the response to *Fg* infection by controlling the expression of CCoAOMT genes in wheat.

Lignin is mainly involved in the basal disease resistance in plants [36, 37]. Furthermore, we validated the expression patterns of 11 selected FHB-responsive TaCCoAOMT genes between mock and Fg infection samples in the resistant RIL genotype R75 and susceptible RIL genotype S98 using the spray inoculation method. Almost all 11 selected TaCCoAOMTs displayed differential expression during *Fg* infection although different change trends were also found between them. At the same time, the expressioin patterns of them detected by qRT-PCR were also consistent with those of RNA-seq analysis, which not only demonstrated the roles of TaCCoAOMTs involved in the response to *Fg* infection, but also provided some candidates for further functional studies.

Finally, we used resequencing data to investigate the genetic variations and divergence of the CCoAOMT family in wheat and its relative populations. Results indicated that wild species showed high genetic diversity and rich haplotype composition in this family compared to cultivated species, suggesting selection effect was exerted on this family and obvious genetic bottleneck has occurred at it during wheat domestication and improvement processes [38]. Wild populations possessed specific haplotypes of the CCoAOMT genes that were lost in cultivated populations, which holds promising for enriching the genetic diversity and also improving the traits controlled by the CCoAOMT genes in cultivated wheat, such as FHB resistance.

## Conclusions

This is the first study to identify the CCoAOMT family in wheat at the genome level. The genomic organization, phylogenetic relationship, exon-intron structure and cis-elements as well as the expression profiles of this family were systematically investigated and characterized. Furthermore, the expression patterns and co-expression network of these genes involved in *Fg* infection were also investigated. providing some useful insights on the roles of *TaCCoAOMTs* in the FHB response and tolerance. Additionally, the genetic diversity and divergence of these CCoAOMT genes in wheat and its relative populations were analysed based on resequencing data. This study not only shed light on the potential function of the CCoAOMT family in regulating wheat lignan biosynthesis and the FHB response but also provided some clues for the evolution of this family in wheat and other plants.

## Methods

### Identification of CCoAOMT genes in wheat

For the identification of the CCoAOMT family in wheat, the protein sequences of the wheat genome were retrieved from the Ensembl plant database to use as the local protein database (ftp://ftp.ensemblgenomes.org/pub/plants/release-50/gff3/triticum_aestivum). CCoAOMT genes in Arabidopsis and rice [25] were used to perform a BLASTP search against the local protein database with the threshold of E-value < 1e^− 5^ [39]. The PFAM profile (PF01596) was downloaded from the PFAM database (https://pfam.xfam.org/) and used as the query to search against the local protein database using HMMER 3.0 with the threshold of E-value < 1e^− 5^. The results of HMMER and BLASTP were integrated together, and the redundant were manually removed. Then, the putative wheat CCoAOMT genes were submitted to SMART (http://smart.embl-heidelberg.de/) and PFAM database (PF01596) (http://pfam.xfam.org/search) to predict the conserved protein domain, and those containing a complete CCoAOMT domain were remained as candidates. The candidate TaCCoAOMT proteins were submitted to the ExPASy database (https://www.ncbi.nlm.nih.gov/Structure/bwrpsb/bwrpsb.cgi) to compute the theoretical isoelectric point (pI) and molecular weight (Mw). The cello tool (http://cello.life.nctu.edu.tw/) was used to predict the subcellular localization. The orthologues of each putative TaCCoAOMT gene with Arabidopsis or rice were determined by BLASTP results and further validated by retrieval in the Ensembl plant database.

### Phylogenetic, gene structure, conserved motif and cis-element analysis of TaCCoAOMTs

Multiple sequence alignment was performed using ClustalX v2.0 [40]. The neighbor-joining method embedded in the MEGA-X program was used to construct the phylogenetic tree, and bootstrapping was set to 1000 [41]. Additionally, conserved motifs of TaCCoAOMT proteins were predicted using MEME v5.2.0 with the default parameters. The gene and motif structures were displayed based on GTF annotation files using TBtools [42]. The upstream 1500 bp region of each TaCCoAOMT gene was extracted and submitted to the PlantCARE database (http://bioinformatics.psb.ugent.be/webtools/plantcare/html/) to predict cis-elements.

### Expression analysis of TaCCoAOMT genes using RNA-seq

A total of 106 RNA-seq samples in different tissues (grain, leaf, root, spike, stem) and under diverse stress treatments (heat, salt, low temperature, drought, *Fg* inoculation) were downloaded from the URGI (http://wheat-urgi.versailles.inra.fr/) and the NCBI database (https://www.ncbi.nlm.nih.gov/) (accession nos. SRP045409, SRP062745, SRP043554 and SRP045409). All of these RNA-seq data were mapped to the wheat reference genome IWSGCv1.1 by STAR v2.7.6a [43] and the fragments per kilobase per million (FPKM) were calculated by StringTie v2.1.2 [44]. The expression patterns were displayed using R featureCounts v2.0.1 [45]and the edgeR package was used for reads normalization, while |log2-fold change| > = 1 and P values < = 0.05 were used as the parameters to identify differential expressed gene. Gene ontology (GO) enrichment was performed using AgriGO v2 (http://systemsbiology.cau.edu.cn/agriGOv2/index.php) and *T. aestivum* was set as the background. The GO enrichment results were plotted by ggplot2 and divided into three classes.

### FHB-related coexpression network construction and miRNA analysis

A co-expression network was constructed using the WGCNA tools based on the 48 RNA-seq samples of four wheat varieties under *Fg* treatment. To obtain the related module and clarify gene interactions, we set the restricted minimum gene number to 30 for each module and used a threshold of 0.25 to merge the similar modules. Genes had higher weights in important modules were chosen to construct a co-expression network. Publicly available trait data, including Fg treatment, Fg time, Fg percent, Fg GAPDH (glyceraldehyde-3-phosphate dehydrogenase) and DON(deoxynivalenol), were used for trait-module correlation analysis [30]. miRNA binding sites were predicted using psRNATarget (http://plantgrn.noble.org/psRNATarget/analysis) with default parameters and all of the wheat miRNAs were used. The regulatory network of the TaCCoAOMT gene and miRNA were visualized using Cytoscape v3.8.0 [46].

### Haplotype and population genetics analysis of TaCCoAOMT

VCF files of wheat resequencing were downloaded from Genome Variation Map (https://bigd.big.ac.cn/gvm) (accession no. GVM000082) [31], which contained the genome variations of a total of 163 bread wheat accessions, 13 durum wheat accessions, 29 domesticated emmer wheat accessions, 28 wild emmer wheat accessions, 29 *T. urartu* accessions and 30 *Aegilops tauschii* accessions. SNPs in the coding region of TaCCoAOMT genes were extracted based on the chromosome location using TBtools. Furthermore, the haplotype organization and frequency were investigated by an in-house Python script.

### Validation of the expression of TaCCoAOMTs through qRT-PCR analysis

For experimental verification, a FHB resistant RIL line (R75) and a FHB susceptible line (S98) from the wheat RIL population developed by single-seed descent from a cross between the susceptible US wheat variety Wheaton and the Chinese resistant wheat landrace HYZ, which were generously provided by Prof. Tao Li, Yangzhou University, China, were used [47]. The plant material was grown in a greenhouse to heading and then a spray inoculation method was used for *Fg* inoculation, following the method as described by Buerstmayr et al. with some modification [48]. A total of 5 inoculated spikes were collected to pool into one sample at 2 days post inoculation (dpi) and three biological replications were adopted. Meanwhile, the counterpart mock samples were collected by the same method. RNA Easy Fast Plant Tissue Kit (Tiangen, Beijing, China) was used to extract total RNA from all samples and RT Master Mix Perfect Real-Time kit (Takara, Dalian, China) was used to synthesize cDNA according to the manufacturer’s instruction. qRT-PCR reaction was performed on a QuantStudioTM 7 Flex System (Thermo Fisher Scientific, USA) using SYBR® Green Premix Pro Taq HS qPCR Kit (Accurate Biology, Hunan, China) with the following thermal cycling conditions: 95 ℃ for 30 s followed by 40 cycles of 95 ℃ for 3 s, 60 ℃ for 30 s. All reactions were performed in three separate technological replicates. The expression levels of these 11 randomly selected TaCCoAOMTs were calculated using the 2^−ΔΔCT^ method with *TaActin2* as the internal reference gene. The primers used in this study are listed in the supplementary file Table S8.

## Supplementary Information

**Additional file 1:** **Figure S1**. Motifs found in* TaCCoAOMT* genes.**Figure S2**. Prediction of the cis-element in promoter regions of *TaCCoAOMT* genes.**Figure S3**. GO enrichment for *TaCCoAOMT* genes.**Figure S4**. Main haplotype and frequency of *TaCCoAOMT* genes in the A subgenome of Triticum.**Figure S5**. Main haplotype and frequency of *TaCCoAOMT* genes in the B subgenome of Triticum.**Figure S6**. Main haplotype and frequency of *TaCCoAOMT*genes in the D subgenome of Triticum.

**Additional file 2:** **Table S1**. Corresponding ID of CCoAOMT genes in Arabidopsis and rice.**Table S2**. Characteristics of cis-acting regulatory elements in the promoter region of CCoAOMT genes in wheat.**Table S3**. GO enrichment analysis of the identified *TaCCoAOMT*s.**Table S4**. Differential expression analysis of *TaCCoAOMT* genes under abiotic stress.**Table S5**. Differential expression analysis of *TaCCoAOMT* genes under *Fg*treatment.**Table S6**. Identification of miRNA bind sites in *TaCCoAOMT* genes.**Table S7**. Haplotype of CCoAOMT genes in five Triticum species.**Table S8**. The list of primers used for qRT-PCR in this study.

## Data Availability

All of the datasets supporting the results of this article are included within the article and its Additional files. And the datasets generated for phylogenetic tree analysis during the current study are available in the Treebase repository, http://purl.org/phylo/treebase/phylows/study/TB2:S28407?x-access-code=96af71ab9297e6ae630774617ab5c9d&format=html.

## Abbreviations

FHB: Fusarium head blight
Fg: *Fusarium graminearum*
CCoAOMT: caffeoyl-coenzyme A O-methyltransferase
TaCCoAOMT: Wheat CCoAOMT
HMM: Hidden Markov Model
CDS: Coding sequence
GO: Gene ontology
qRT-PCR: Quantitative real-time polymerase chain reaction
