# Supplementary material for: Genome-wide identification and characterization of caffeoyl-coenzyme A O-methyltransferase genes related to the Fusarium head blight response in wheat
Source: BMC Genomics. 2021 Jul 4;22:504. doi: 10.1186/s12864-021-07849-y (PMC8254967; doi:10.1186/s12864-021-07849-y)
Supplement: Supplementary file 1 — Additional file 1: Figure S1. Motifs found in TaCCoAOMT genes.Figure S2. Prediction of the cis-element in promoter regions of TaCCoAOMT genes.Figure S3. GO enrichment for TaCCoAOMT genes.Figure S4. Main haplotype and frequency of TaCCoAOMT genes in the A subgenome of Triticum.Figure S5. Main haplotype and frequency of TaCCoAOMT genes in the B subgenome of Triticum.Figure S6. Main haplotype and frequency of TaCCoAOMTgenes in the D subgenome of Triticum. [file 12864_2021_7849_MOESM1_ESM.docx]

**Genome-wide identification and characterization of caffeoyl-coenzyme A O-methyltransferase genes related to the Fusarium head blight response in wheat**

Guang Yang^1#^, Wenqiu Pan^1#^, Ruoyu Zhang^1^, Yan Pan^1^, Qifan Guo^1^, Weining Song^1,2^,Weijun Zheng^1*^, Xiaojun Nie^1,2*^

^1^State Key Laboratory of Crop Stress Biology in Arid Areas, College of Agronomy and Yangling Branch of China Wheat Improvement Center, Northwest A&F University, Yangling 712100, Shaanxi, China

^2^ICARDA-NWSUAF Joint Research Centre, Northwest A&F University,Yangling712100, Shaanxi, China

*Corresponding author:

Weijun Zheng: zhengweijun@nwafu.edu.cn; Xiaojun Nie: small@nwsuaf.edu.cn

**Supplementary figure legends**

**Figure S1. Motifs found in *TaCCoAOMT* genes.**

**Figure S2.** **Prediction of the cis-element in promoter regions of *TaCCoAOMT* genes.**

**Figure S3. GO enrichment for *TaCCoAOMT* genes.**

**Figure S4. Main haplotype and frequency of *TaCCoAOMT* genes in the A subgenome of Triticum.**

**Figure S5. Main haplotype and frequency of *TaCCoAOMT* genes in the B subgenome of Triticum.**

**Figure S6. Main haplotype and frequency of *TaCCoAOMT* genes in the D subgenome of Triticum.**


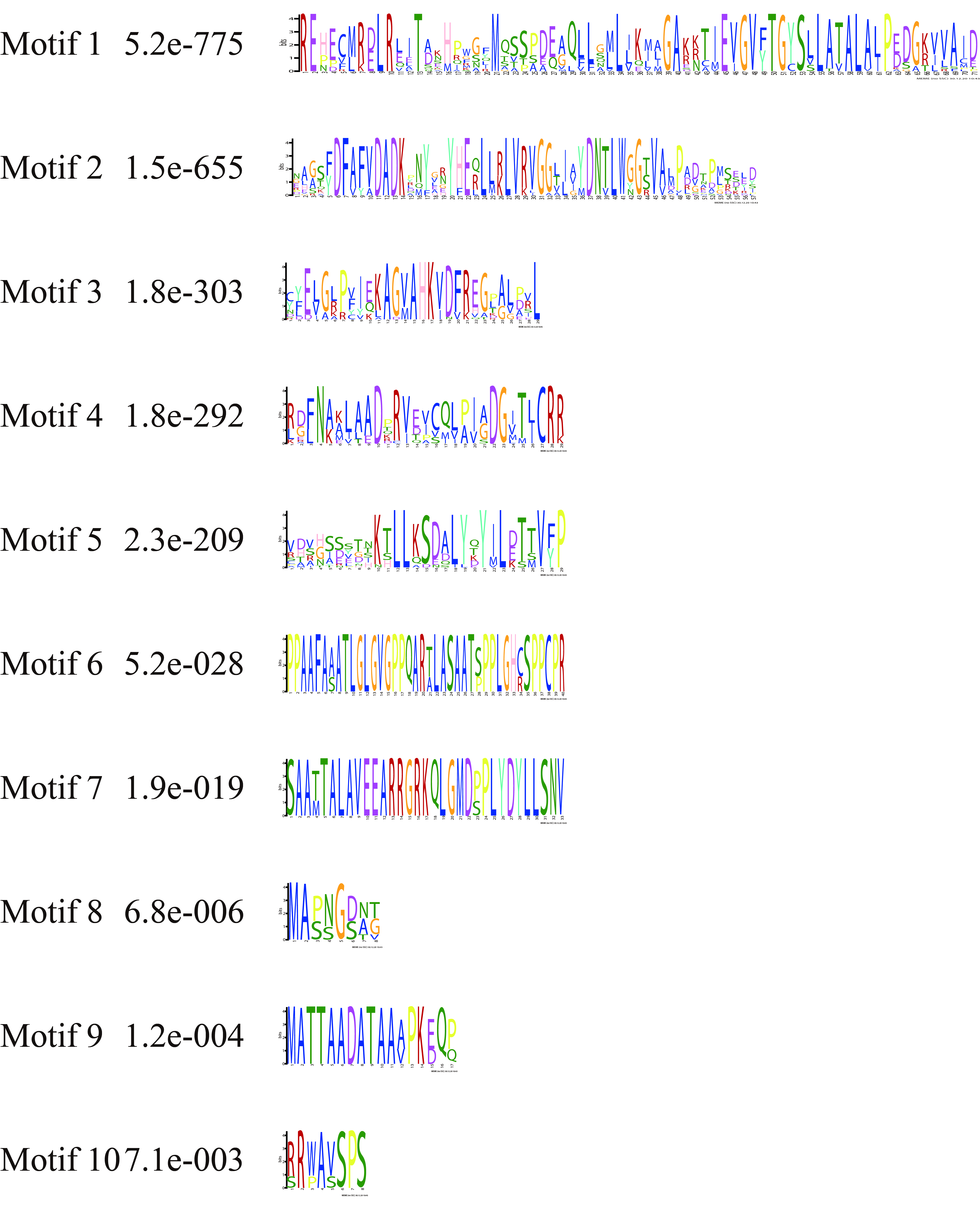


**Figure S1. Motifs found in *TaCCoAOMT* genes.**


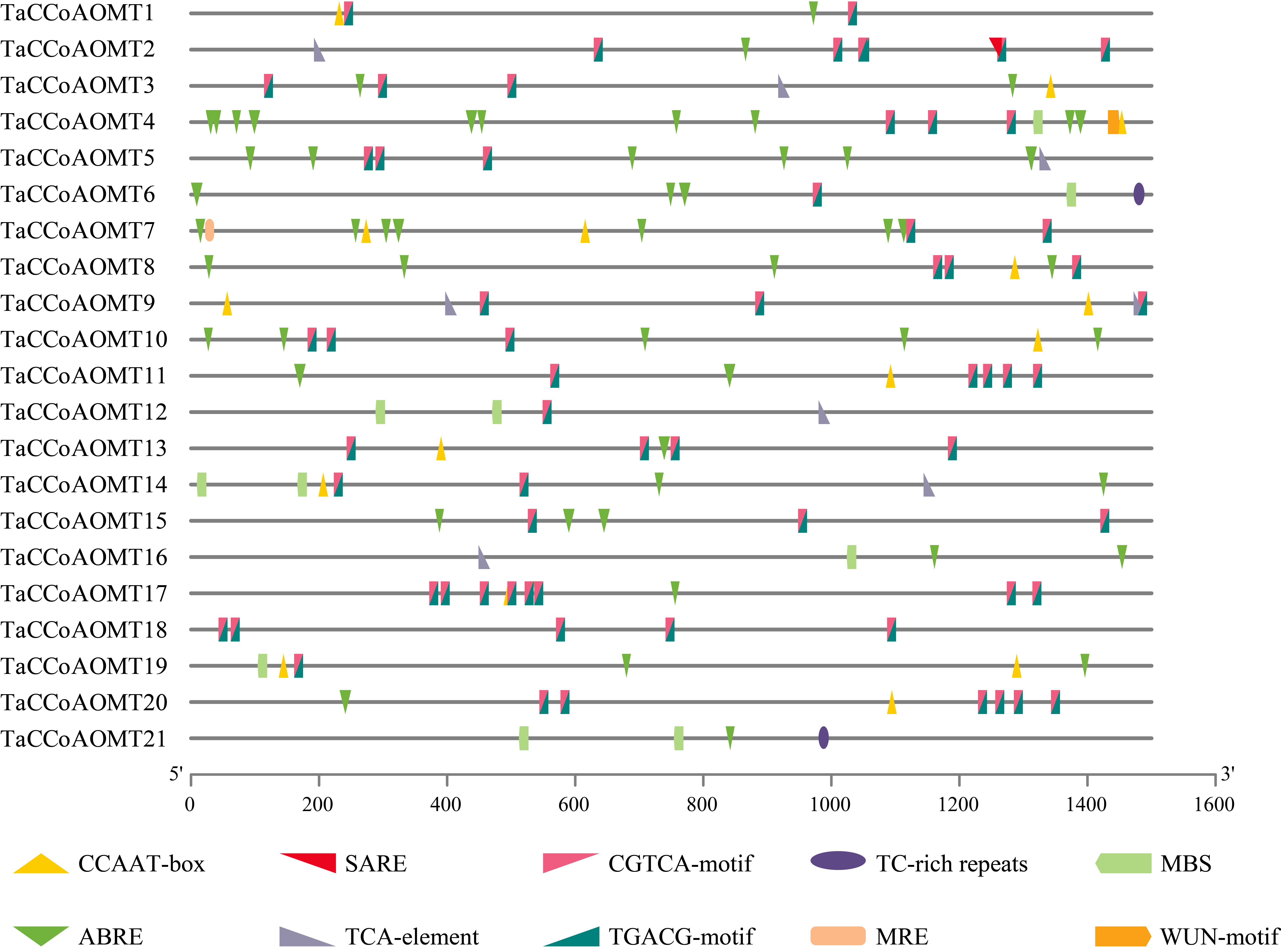


**Figure S2. Predicted cis-element analysis in promoter regions of *TaCCoAOMT* genes.**


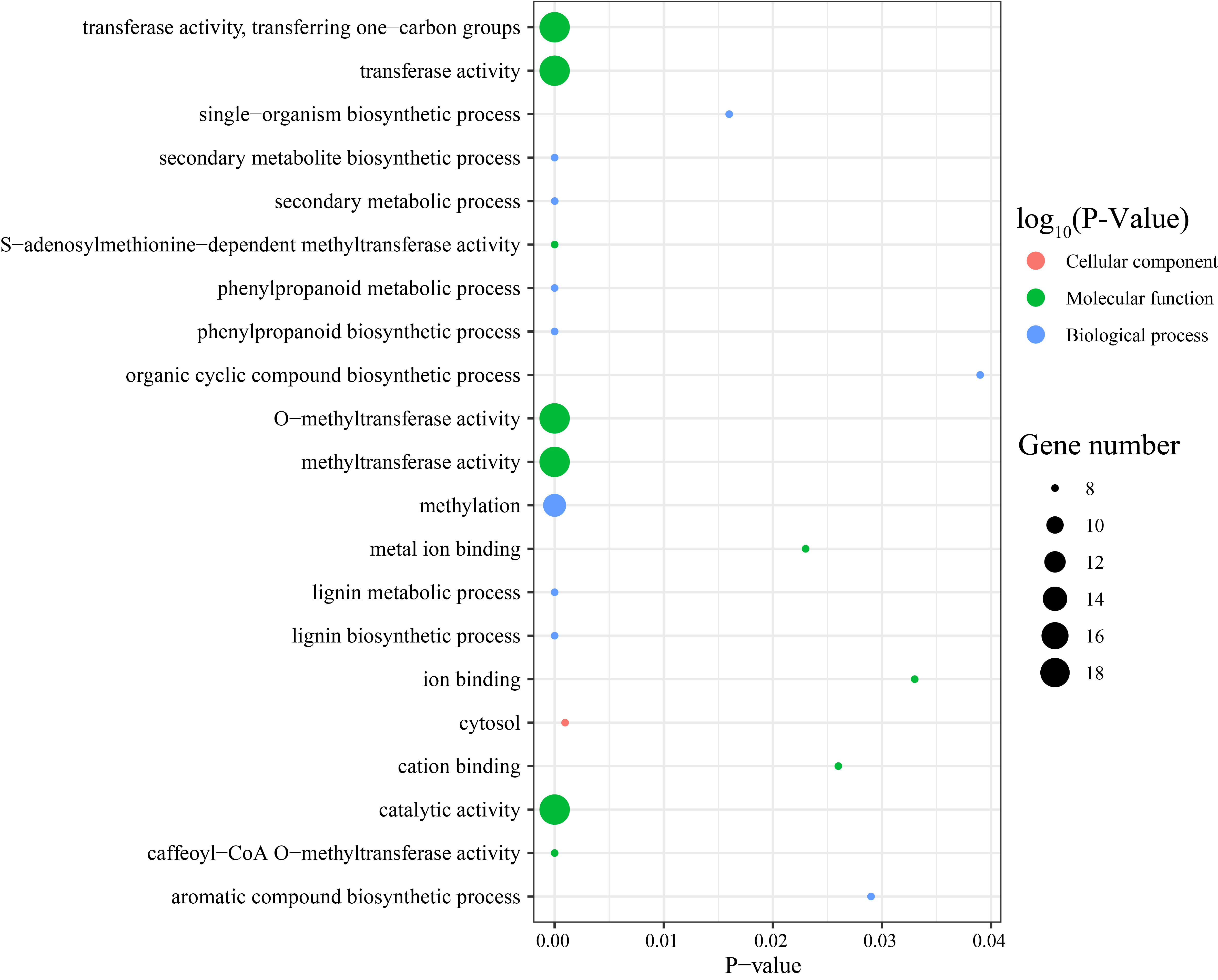


**Figure S3. GO enrichment for *TaCCoAOMT* genes.**


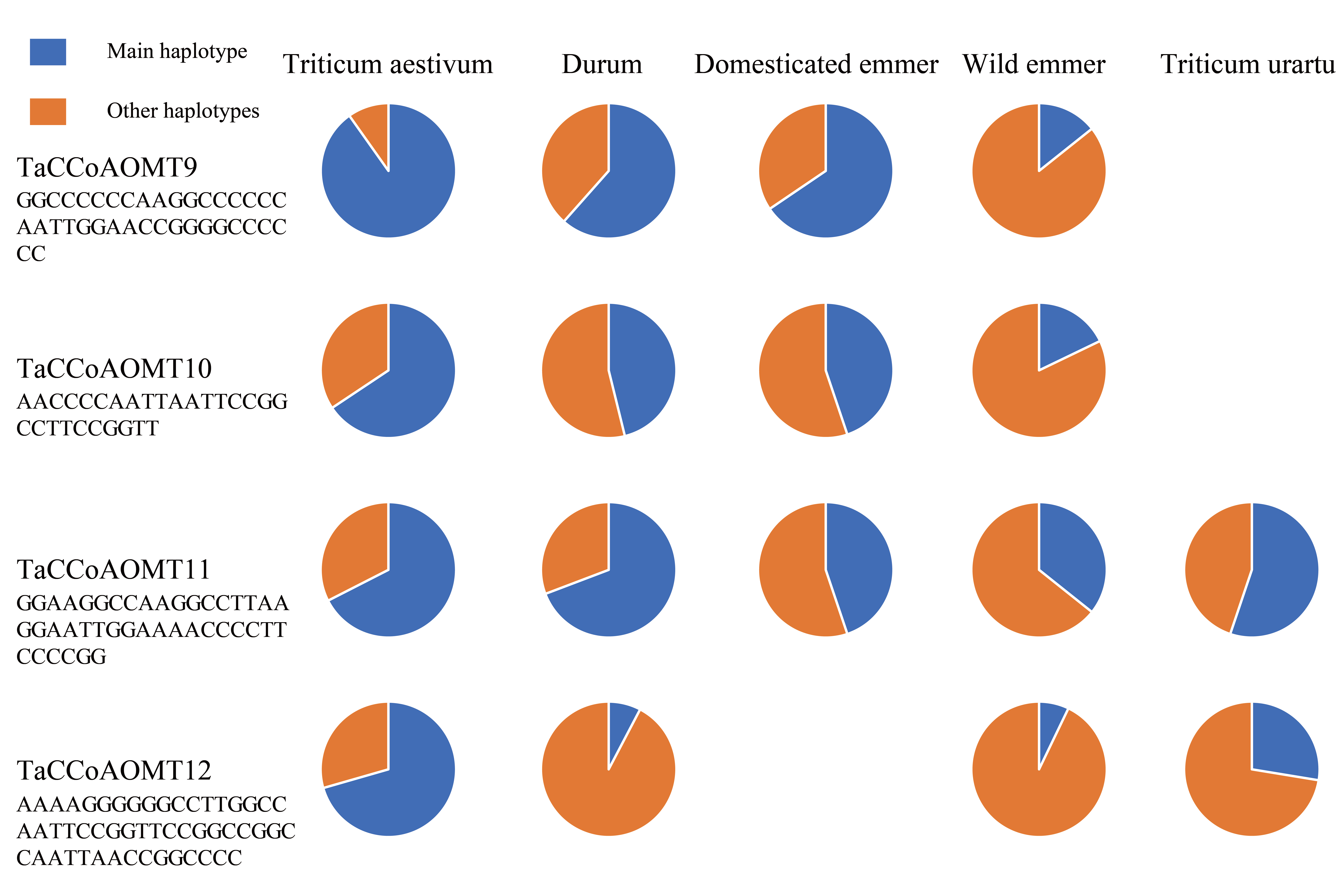


**Figure S4. Main haplotype and frequency of TaCCoAOMT genes in the A subgenome of Triticum.**


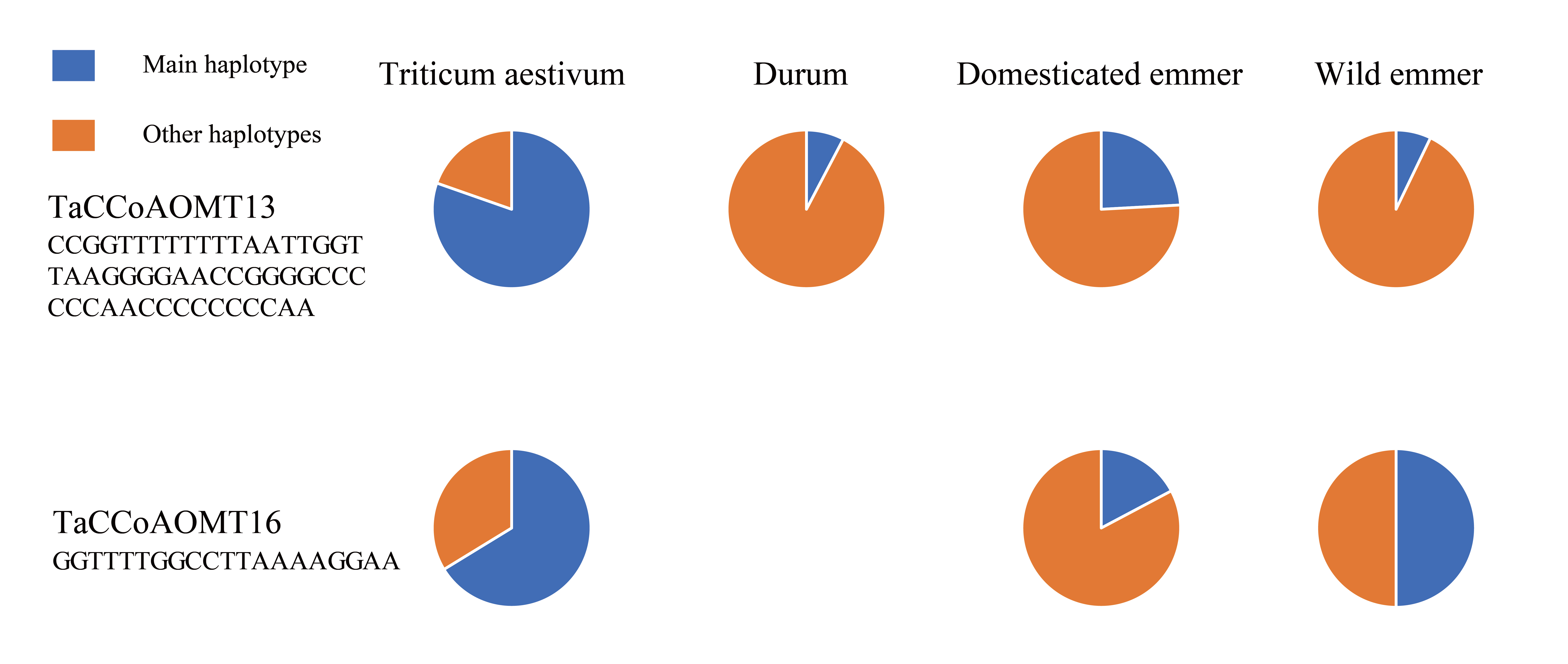


**Figure S5. Main haplotype and frequency of TaCCoAOMT genes in B the subgenome of Triticum.**


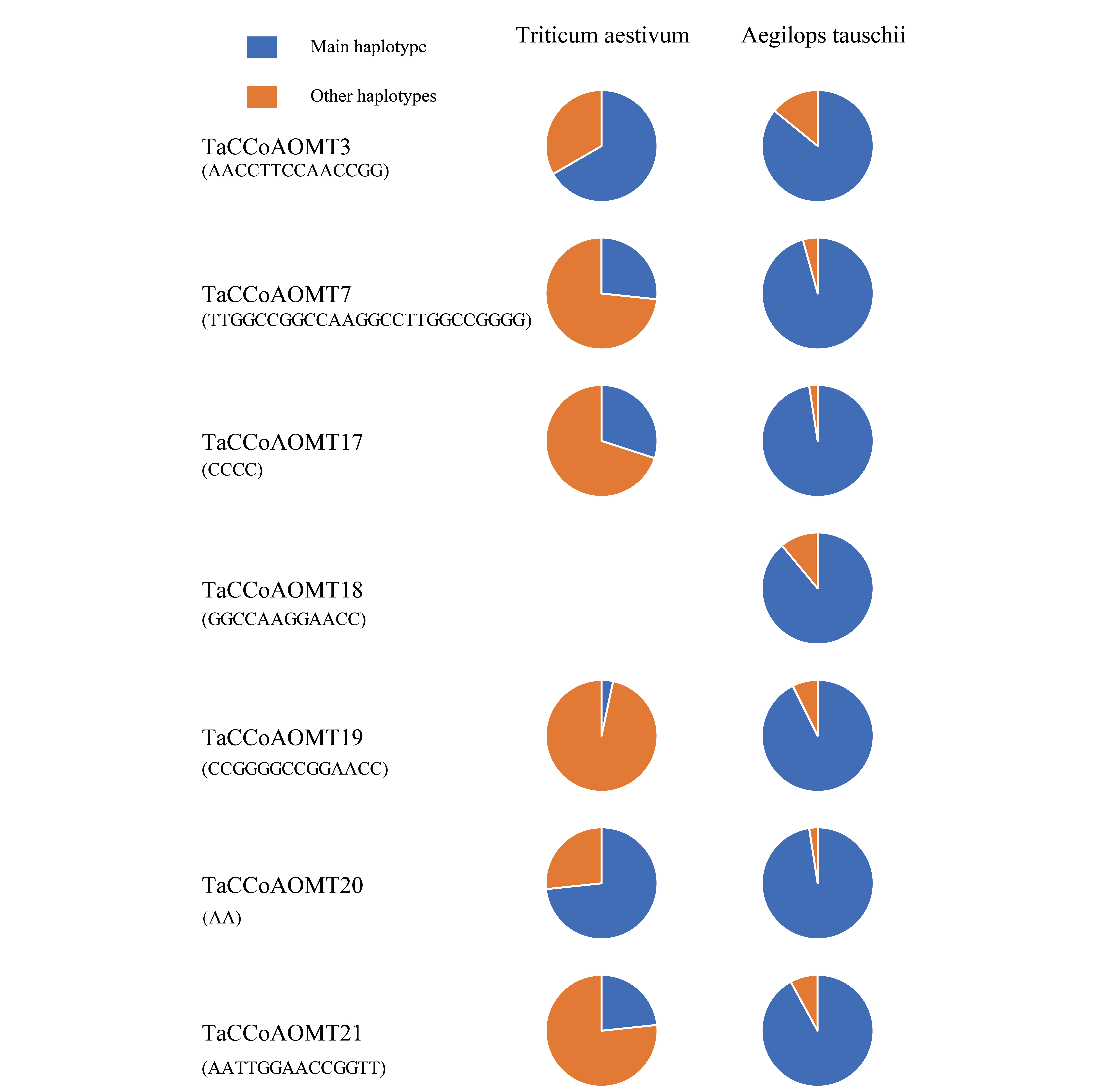


**Figure S6. Main haplotype and frequency of TaCCoAOMT genes in the D subgenome of Triticum.**
